# A False-Negative Newborn Screen for Tyrosinemia Type 1—Need for Re-Evaluation of Newborn Screening with Succinylacetone

**DOI:** 10.3390/ijns9040066

**Published:** 2023-12-04

**Authors:** Allysa M. Dijkstra, Kimber Evers-van Vliet, M. Rebecca Heiner-Fokkema, Frank A. J. A. Bodewes, Dennis K. Bos, József Zsiros, Koen J. van Aerde, Klaas Koop, Francjan J. van Spronsen, Charlotte M. A. Lubout

**Affiliations:** 1 Section of Metabolic Diseases, Beatrix Children’s Hospital, University of Groningen, University Medical Center Groningen, 9700 RB Groningen, The Netherlands; a.m.dijkstra@umcg.nl (A.M.D.); k.van.vliet@umcg.nl (K.E.-v.V.); f.j.van.spronsen@umcg.nl (F.J.v.S.); 2 Laboratory of Metabolic Diseases, Department of Laboratory Medicine, University of Groningen, University Medical Center Groningen, 9700 RB Groningen, The Netherlands; m.r.heiner@umcg.nl; 3 Section of Pediatric Gastroeneterology and Hepatology, Beatrix Children’s Hospital, University of Groningen, University Medical Center Groningen, 9700 RB Groningen, The Netherlands; f.a.j.a.bodewes@umcg.nl; 4 Department of Genetics, University of Groningen, University Medical Center Groningen, 9700 RB Groningen, The Netherlands; d.k.bos@umcg.nl; 5 Princess Máxima Center for Pediatric Oncology, 3584 CX Utrecht, The Netherlands; j.zsiros@prinsesmaximacentrum.nl; 6 Department of Pediatric Infectious Disease and Immunology, Amalia’s Children Hospital, Radboud University Medical Center, 6525 GA Nijmegen, The Netherlands; koen.vanaerde@radboudumc.nl; 7 Section Metabolic Diseases, Department of Pediatrics, University Medical Center Utrecht, 3584 CX Utrecht, The Netherlands; k.koop@umcutrecht.nl

**Keywords:** tyrosinemia type 1, succinylacetone, cirrhosis, hepatocellular carcinoma, newborn screening

## Abstract

Undiagnosed and untreated tyrosinemia type 1 (TT1) individuals carry a significant risk for developing liver fibrosis, cirrhosis and hepatocellular carcinoma (HCC). Elevated succinylacetone (SA) is pathognomonic for TT1 and therefore often used as marker for TT1 newborn screening (NBS). While SA was long considered to be elevated in every TT1 patient, here we present a recent false-negative SA TT1 screen. A nine-year-old boy presented with HCC in a cirrhotic liver. Additional tests for the underlying cause unexpectedly revealed TT1. Nine years prior, the patient was screened for TT1 via SA NBS with a negative result: SA 1.08 µmol/L, NBS cut-off 1.20 µmol/L. To our knowledge, this report is the first to describe a false-negative result from the TT1 NBS using SA. False-negative TT1 NBS results may be caused by milder TT1 variants with lower SA excretion. Such patients are more likely to be missed in NBS programs and can be asymptomatic for years. Based on our case, we advise TT1 to be considered in patients with otherwise unexplained liver pathology, including fibrosis, cirrhosis and HCC, despite a previous negative TT1 NBS status. Moreover, because the NBS SA concentration of this patient fell below the Dutch cut-off value (1.20 µmol/L at that time), as well as below the range of cut-off values used in other countries (1.29–10 µmol/L), it is likely that false-negative screening results for TT1 may also be occurring internationally. This underscores the need to re-evaluate TT1 SA NBS programs.

## 1. Introduction

In tyrosinemia type 1 (TT1; OMIM #276700), a rare inherited metabolic disease (IMD) of tyrosine (Tyr) catabolism, toxic metabolites such as fumarylacetoacetate and succinylacetone (SA) accumulate due to a deficiency of the fumarylacetoacetate hydrolase (FAH) enzyme. Untreated, TT1 can cause liver pathology including acute liver failure, liver fibrosis, cirrhosis, hepatocellular carcinoma (HCC) or hepatoblastoma, as well as renal tubular dysfunction, episodes of porphyria crises with neuropathy, and suboptimal neurocognitive outcomes [1,2].

Before the current TT1 treatment became available, patients often required liver transplantation [1,3]. However, early diagnosis, monitoring by measurement of SA, and treatment with 2-(2-nitro-4-trifluoromethylbenzoyl)-1,3-cyclohexanedione (NTBC) have greatly improved the outcome for TT1 patients [4]. Therefore, TT1 was added to the Dutch newborn screening (NBS) program in 2007, using dried blood spot (DBS) Tyr as a screening marker with a cut-off of 500 µmol/L [5,6]. However, many false-positive results emerged, resulting in the program’s termination soon after its introduction. Thereafter, Tyr was no longer used as marker in the Dutch screening for TT1.

In October 2008, TT1 NBS was re-introduced, this time using DBS SA as marker with a cut-off of 1.50 µmol/L, measured using the PerkinElmer NeoBase assay on liquid chromatography tandem mass spectrometry (LC-MS/MS) [7,8,9]. To improve the sensitivity of the program, the cut-off was lowered from 1.50 µmol/L to 1.20 µmol/L in 2009. In 2018, the assay and mass spectrometer changed to PerkinElmer NeoBase2 on a Waters Xevo TQD mass spectrometer. The limit of quantification (LOD) for the new method was lower, causing a change in the cut-off from 1.20 to 0.90 µmol/L to further improve sensitivity. After implementation, the LOD turned out to be even lower, because of which the cut-off changed to 0.6 µmol/L in 2019.

Although DBS SA is reported to have high positive and negative predictive values for TT1 [10], false-positive results are often observed in the Dutch TT1 NBS [11,12]. In addition, we recently encountered a historical false-negative TT1 SA NBS result in a nine-year-old patient presenting with HCC in a cirrhotic liver. False-negative results have been reported previously for the TT1 screening programs using Tyr as marker, but, to the best of our knowledge, not using SA [13,14,15,16].

In this report, we aim to create awareness that TT1 patients can be missed despite a negative SA TT1 NBS result depending on the cut-off used. Based on our experience we advise TT1 to be considered in any patient with otherwise unexplained liver pathology including liver fibrosis, cirrhosis, liver failure, and HCC. Additionally, we advocate for an assessment of the NBS program using SA.

## 2. Case Presentation

A nine-year-old boy was admitted to the hospital with a presumptive diagnosis of acute appendicitis. However, the ultrasound showed a liver mass, most likely to be of infectious cause. The MRI revealed a circumscriptive lesion with a diameter of 45 mm in segment 4 of the liver. The serum alpha-fetoprotein (AFP) concentration was markedly increased, i.e., 66,000 µg/L (reference value 0.8–4.5 µg/L), which strengthened the suspicion of a liver tumour, presumably HCC or hepatoblastoma. The fluorodeoxyglucose (FDG) PET/CT showed increased glucose consumption in the liver, and a liver biopsy confirmed the presence of a liver tumour which was difficult to classify due to the necrotic tissue in the sample, but was favoured as an embryonal hepatoblastoma, or, based on the TERT promotor mutation found in the tumour, a hepatocellular neoplasm not otherwise specified (HCN-NOS).

After chemotherapy, which resulted in a stable disease but no significant tumour shrinkage, the patient received a living donor liver transplantation at the University Medical Center Groningen (UMCG), in the Netherlands, three months after primary diagnosis.

Post hepatectomy pathological tumour evaluation favoured HCC, and also revealed severe (septal) fibrosis in parts of the liver that could not be explained as chemotherapy toxicity or autoimmune hepatitis. No lymph node metastases were found.

The atypical presentation of HCC and severe liver fibrosis led to the decision to perform additional genetic analysis. Whole exome sequencing (WES, Agilent SureSelect Human All exome V7) followed by analysis of genes associated with liver diseases revealed two compound heterozygous variants in *FAH*, namely the pathogenic variant c.1062 + 5G > A and the likely pathogenic variant c.696C > T, p.(Asn232=), consistent with TT1.

TT1 was biochemically confirmed through measurements of SA and Tyr concentrations in stored pre-transplantation blood samples. Plasma Tyr was elevated (433 µmol/L, reference range 33–106 µmol/L) and plasma phenylalanine was within the normal range (67 µmol/L, reference range 35–89 µmol/L). Plasma SA was also elevated (0.23 µmol/L, reference range <LOQ (0.0013 µmol/L). SA concentrations were likely to be even higher at the time of sampling, as the sample had been stored for five months, during which it had gone through multiple freeze-thaw cycles at −20 °C. SA is known to degrade significantly, with losses up to 50% within 24 h at room temperature and up to 60% within four weeks at −20 °C [17].

Since his liver transplantation, the patient is doing well. The Tyr concentrations have normalized (+/−100 µmol/L). The SA concentrations remained mildly elevated in the urine (+/−0.079 mg/mmol creatinine), but not in the DBS (SA < reference cut-off), similar to findings of Pierik et al. in which urinary SA excretion in nine patients post liver transplantation ranged from 0.1 to 5.1 mg/mmol creatinine [18]. Additional treatment with a low phenylalanine–tyrosine diet and/or NTBC were deemed unnecessary, and the patient receives a low frequency follow-up once a year (combined hepatology and metabolic paediatrics). The patient is monitored for tubular dysfunction, which he has not shown to date.

Surprisingly, prior to developing HCC, our patient did not show the classic signs of untreated TT1; he did not experience TT1 symptoms such as bleeding tendency, feeding problems, failure to thrive, enlarged abdomen, renal tubulopathy, or porphyria crises although his presentation of acute abdominal pain may have been part of an intermittent porphyria attack due to TT1. Furthermore, overall his intellectual development was normal, and he performed well at school.

The unusual late diagnosis of TT1 prompted us to revisit the patient’s nine-year-old NBS results, which were obtained from the Dutch National Institute for Public Health and the Environment (RIVM). The NBS heel prick SA concentration was 1.08 µmol/L (Tyr 288 µmol/L), which was below the TT1 SA cut-off of 1.20 µmol/L at the time, confirming a false-negative TT1 SA NBS result in this patient.

## 3. Discussion

We presented a nine-year-old boy with HCC in a cirrhotic liver who was unexpectedly diagnosed with TT1 after receiving a negative SA NBS result nine years prior. To our knowledge, this is the first report of a false-negative result from the TT1 NBS using SA.

Our patient’s lack of TT1-related symptoms before developing HCC suggests he suffered from a milder form of TT1. TT1 is a disease with large heterogeneity in clinical presentation, varying between TT1 families and relatives without clear genotype–phenotype correlations [1]. Therefore, disease severity is traditionally classified according to the age of onset of clinical symptoms, with the milder phenotypes presenting at >6 months [19]. Nowadays, the outcome is mainly dependent on NTBC treatment, which gives a significantly better prognosis in children treated pre-symptomatically compared to those starting treatment later [4,20].

Although rare, mild TT1 phenotypes have been reported [21,22,23,24]. These phenotypes can be the result of a gene reversion, meaning spontaneous self-correction of the germline pathogenic *FAH* variant with partial restoration of hepatocyte FAH activity [19,22,25]. Gene reversion is likely caused by mutation pressure (caused by e.g., SA and fumarylacetoacetate) combined with high levels of cell proliferation and can be seen as hepatocyte mosaicism upon immunostaining [23]. The restored ‘normal hepatocytes’ have a selective growth advantage over pathogenic hepatocytes, as only FAH defected cells are sensitive to fumarylacetoacetate induced apoptosis [26,27]. Gene reversion occasionally occurs in the common c.1062 + 5G > A variant (one of our patient’s variants), which affects splicing by preventing usage of the exon 12 5′splice site [27,28].

Rarely, FAH activity is restored by the coexistence of the c.1062 + 5G > A variant and the rare c.1061C > A *FAH* variant on the same allele [28]. This results in rescuing of the defective splice site and increased *FAH* transcription up to 60%. Our patient does not have the c.1061C > A variant. However, the DNA testing was performed in blood samples, not in liver cells. Therefore, we cannot exclude that splice site rescue of gene reversion (with liver mosaicism) is at play.

Additionally, Cassiman et al. and Blackburn et al. describe three patients with mild TT1 phenotypes in the absence of gene reversion. These patients, who were homozygous for the previously unknown c.103G > A (p.Ala35Thr) and c.424A > G (p.Arg142Gly) variants, respectively, developed severe liver disease at young ages [23,24]. One of them was diagnosed because of bleeding episodes at four months (Cassiman et al.) [23], and the other two were diagnosed after hepatosplenomegaly, fibrosis and HCC at age 12 and 13 (Blackburn et al.) [24]. SA was undetectable in the blood and urine, and the enzymatic studies showed low, but not absent liver FAH activity. DNA sequencing of the variants in the liver tissue did not show any form of mosaicism and thus gene reversion [24]^.^

Next to the c.1062 + 5G > A variant, our patient has the likely pathogenic c.696C > T, p.(Asn232=) variant on the other allele. This variant has only been described once and is considered to cause skipping of exon 8 [29]. Surprisingly, we found the same heterozygous variant (in combination with the pathogenic c.674T > G, p.(Ile225Ser) variant on the other allele) in another Dutch TT1 patient who was diagnosed through NBS with a slightly elevated SA concentration of 0.99 µmol/L (cut-off 0.60 µmol/L). This patient only requires low doses of NTBC to keep SA concentrations undetectable (1 mg/kg twice per week). These cases suggest that the c.696C > T, p.(Asn232=) variant may cause milder TT1 phenotypes, and together with the cases of Cassiman et al. and Blackburn et al. [23,24], they highlight that other still largely unknown phenomena can underlie the mild phenotypes of TT1.

Because DBS SA in mild TT1 phenotypes can be either normal or only slightly elevated, patients are likely to be missed in current NBS programs. However, they may still be at risk of developing liver disease later on [21,23,24]. Early diagnosis through adequate NBS is thus of great importance in these ‘mild’ patients. Nonetheless, NBS is complicated by the fact that SA measurements and cut-offs heavily depend on used analytical methods, which vary worldwide, creating bias between measurements and results [10,30]. Regardless, the NBS SA concentration of our patient (1.08 µmol/L) fell below the Dutch cut-off, being 1.20 µmol/L (at that time), and well below the range of cut-offs used in other countries (1.29–10 µmol/L) [10]. This not only underscores the need to re-evaluate the TT1 SA NBS programs, but also shows the likelihood that false-negative TT1 screening results may also be occurring internationally.

Suggestively, screening for TT1 could be improved by adjusting the cut-off for SA [31]. The SA concentration of our patient suggests that cut-offs for many programs should be lowered, which was already done in the Netherlands, reducing the cut-off to 0.60 µmol/L [11,12]. While we do not know yet whether this prevented false-negative results, we did face increases in false-positive results [11,12]. Further lowering the cut-off might aggravate this, which should be taken into account when evaluating NBS programs for TT1.

With regards to the false-negative results worldwide, it is interesting that this paper is the first to report a false-negative screen from the TT1 SA NBS, given the range of SA cut-offs internationally. It may be that other false-negative results are simply underreported. However, as patients with mild forms of TT1 can remain symptom-free for years, it is plausible that (some of) such patients have not yet developed symptoms or are treated for HCC without having proper diagnostic (including genetic) investigations, thus escaping diagnosis of TT1. Making this more likely is that many countries only included TT1 as part of their extended NBS programs somewhere between the early 2000′s until just recently [32]. Consequently, we urge everyone to consider TT1 in patients with otherwise unexplained liver pathology, including fibrosis, cirrhosis and HCC despite a previous negative TT1 NBS status.

## 4. Conclusions

Undiagnosed and untreated tyrosinemia type 1 (TT1) individuals carry a significant risk for the development of liver disease. However, patients with mild forms of TT1 might be missed in the NBS for TT1 and can go years without developing clinical symptoms. Based on our experience, we advise TT1 to be considered in any patient with otherwise unexplained liver pathology including liver fibrosis, cirrhosis, liver failure, and HCC despite previous NBS status.

## Data Availability

Data is not available due to privacy restrictions.

## Abbreviations

AFP	alpha-fetoprotein
DBS	dried blood spot
FAH	fumarylacetoacetate hydrolase
FDG	Fluorodeoxyglucose
HCC	hepatocellular carcinoma
IMD	inherited metabolic disease
NBS	Newborn screening
NTBC	2-(2-nitro-4-trifluoromethylbenzoyl)-1,3-cyclohexanedione
SA	succinylacetone
TT1	tyrosinemia type 1
Tyr	tyrosine
